# Development and Validation of an Improved PCR Method Using the 23S-5S Intergenic Spacer for Detection of Rickettsiae in Dermacentor variabilis Ticks and Tissue Samples from Humans and Laboratory Animals

**DOI:** 10.1128/JCM.02605-15

**Published:** 2016-03-25

**Authors:** Madhavi L. Kakumanu, Loganathan Ponnusamy, Haley T. Sutton, Steven R. Meshnick, William L. Nicholson, Charles S. Apperson

**Affiliations:** a Department of Entomology, North Carolina State University, Raleigh, North Carolina, USA; b Department of Epidemiology, Gillings School of Global Public Health, University of North Carolina, Chapel Hill, North Carolina, USA; c Rickettsial Zoonoses Branch, National Center for Emerging and Zoonotic Infectious Diseases, Centers for Disease Control and Prevention, Atlanta, Georgia, USA; d Comparative Medicine Institute, North Carolina State University, Raleigh, North Carolina, USA

## Abstract

A novel nested PCR assay was developed to detect Rickettsia spp. in ticks and tissue samples from humans and laboratory animals. Primers were designed for the nested run to amplify a variable region of the 23S-5S intergenic spacer (IGS) of Rickettsia spp. The newly designed primers were evaluated using genomic DNA from 11 Rickettsia species belonging to the spotted fever, typhus, and ancestral groups and, in parallel, compared to other Rickettsia-specific PCR targets (*ompA*, *gltA*, and the 17-kDa protein gene). The new 23S-5S IGS nested PCR assay amplified all 11 Rickettsia spp., but the assays employing other PCR targets did not. The novel nested assay was sensitive enough to detect one copy of a cloned 23S-5S IGS fragment from “Candidatus Rickettsia amblyommii.” Subsequently, the detection efficiency of the 23S-5S IGS nested assay was compared to those of the other three assays using genomic DNA extracted from 40 adult Dermacentor variabilis ticks. The nested 23S-5S IGS assay detected Rickettsia DNA in 45% of the ticks, while the amplification rates of the other three assays ranged between 5 and 20%. The novel PCR assay was validated using clinical samples from humans and laboratory animals that were known to be infected with pathogenic species of Rickettsia. The nested 23S-5S IGS PCR assay was coupled with reverse line blot hybridization with species-specific probes for high-throughput detection and simultaneous identification of the species of Rickettsia in the ticks. “Candidatus Rickettsia amblyommii,” R. montanensis, R. felis, and R. bellii were frequently identified species, along with some potentially novel Rickettsia strains that were closely related to R. bellii and R. conorii.

## INTRODUCTION

Rickettsia species are Gram-negative, obligate intracellular bacteria vectored by a diverse array of arthropods (1). These bacteria are broadly categorized into the spotted fever group (SFG), the typhus group (TG), and two ancestral groups of rickettsiae (2). Rickettsiae are noteworthy because of their global distribution and the large array of species identified as human pathogens (3). Ticks are the principal vectors of SFG rickettsiae, and in particular, Dermacentor variabilis Say, the American dog tick, is an established vector of Rickettsia rickettsii, the causative organism of Rocky Mountain spotted fever (RMSF). Reported cases of SFG rickettsioses (including RMSF) have escalated in the United States in recent years, with a concomitant decrease in the case fatality rate (4). Consequently, the increase in reported cases of illness could be caused by less virulent strains of R. rickettsii (5, 6). However, recent studies of Rickettsia prevalence in D. variabilis have either failed to detect R. rickettsii (7–11) or found it to be exceedingly rare (12, 13). Accordingly, it is possible that mildly pathogenic Rickettsia strains, some of which may be novel species (4, 14–16), are responsible for the escalation of reported cases. To understand the role of novel pathogens in driving the recent increase in SFG rickettsioses, investigations of the diversity of Rickettsia species in D. variabilis ticks should be carried out (17).

PCR-based assays are commonly used for surveillance of rickettsial pathogens in field-collected ticks (18). The 17-kDa protein gene, *ompA*, *ompB*, *SCA4*, and *gltA* are some of the commonly targeted genes for detection of Rickettsia species infection in ticks (8, 13, 19–21). Recently, the 23S-5S intergenic spacer (IGS) has been reported to be a robust target for detection of Rickettsia (22–24). The 23S-5S IGS is a noncoding region of DNA that is present in Rickettsia species (25, 26). The 23S-5S IGS has conserved ends and hypervariable central regions exhibiting sequence diversity, making it an additional target for identification of Rickettsia DNA to the species level (22). While PCR assays targeting different regions of the Rickettsia genome have been used successfully to detect rickettsiae in environmental samples, inconsistencies in the amplification rates of different gene targets in the same samples is not uncommon (8, 23). This problem could potentially result from a lack of specificity of the primers for potentially novel species or to low copy numbers of the target genes.

The objective of our study was to develop a new set of primers for a nested 23S-5S IGS PCR assay that would increase the efficiency of amplification of Rickettsia spp. even at low abundance. We then coupled the 23S-5S IGS PCR assay with reverse line blot (RLB) hybridization for high-throughput screening and simultaneous identification of Rickettsia in ticks.

## MATERIALS AND METHODS

### Primer design and PCR optimization for the 23S-5S IGS.

A set of new primers were designed for a nested PCR assay targeting an ∼350-bp DNA fragment from the 23S-5S IGS region of Rickettsia. The 23S-5S IGS sequences of some Rickettsia spp. (including SFG and ancestral groups) were retrieved from GenBank and aligned using the Clustal W program (27). We used the MEGA version 6 software package (28) to identify 20- to 22-bp regions that were conserved among all groups of Rickettsia. The newly designed nested-PCR primers were RCK/23-5N1F (5′ TGTGGAAGCACAGTAATGTGTG 3′) and RCK/23-5N1R (5′ TCGTGTGTTTCACTCATGCT 3′). The amplification conditions of the thermocycler were optimized by conducting temperature gradient PCR on genomic DNA (gDNA) of some known Rickettsia species (positive-control DNA) and on tick samples that were previously identified as positive for rickettsiae. After optimization, assays were conducted on genomic DNA from 11 known species of Rickettsia belonging to the SFG and the typhus and ancestral groups (Table 1). The DNAs for the Rickettsia spp. were obtained from the Rickettsial Zoonoses Branch, Centers for Disease Control and Prevention (Atlanta, GA, USA), except for *R*. monacensis, for which cell culture material was obtained from Fuller Laboratories (Fullerton, CA, USA).

**TABLE 1:** Amplification of four gene/IGS targets of 11 Rickettsia species with an initial template (gDNA) concentration of 2 ng/μl

Positive-control species (strain)	PCR amplification^*a*^			
	17-kDa nested	*gltA* nested	*ompA* seminested	23S-5S IGS nested
“Candidatus Rickettsia amblyommii” (Darkwater)	+	+	+	+
R. bellii (369C)	−	−	−	+
R. canadensis (2678 [McKeil])	−	+	−	+
R. conorii (Moroccan)	+	+	+	+
R. massiliae (Mtu1)	+	+	+	+
R. monacensis (unknown strain)	+	+	+	+
R. montanensis (Tick)	+	+	+	+
R. parkeri (Maculatum 20)	+	+	+	+
R. rhipicephali (3-7-♀-6)	+	+	+	+
R. rickettsii (Sheila Smith)	+	+	+	+
R. typhi (Wilmington)	+	+	−	+

a +, amplification; −, no amplification.

Primary PCR amplifications were conducted in a 20-μl reaction mixture consisting of 1 μl of genomic DNA (2 ng), 10 μl of AmpliTaq Gold PCR master mix (catalog no. 4398881; Life Technologies, USA), 1 μl of primer RCK/23-5-F (10 μM), 1 μl of primer RCK/23-5-R (10 μM) (22), and 7 μl of nuclease-free water. The nested-PCR mixture consisted of 1 μl of primary amplicons as template DNA, 10 μl of AmpliTaq Gold PCR master mix, 1 μl of primer RCK/23-5N1F (10 μM), 1 μl of primer RCK/23-5N1R (10 μM), and 7 μl of nuclease-free water. Biotin-modified (5′ end) nested primers were used if the amplicons were to be used in RLB hybridization reactions. The PCR conditions used for amplification of 23S-5S IGS fragments were as follows: 95°C for 10 min, 35 cycles of 94°C for 30 s, 60°C for 30 s, and 65°C for 1.5 min, and a final cycle of 65°C for 7 min (for the primary reaction; modified protocol of Lee et al. [23]) and 95°C for 10 min, 30 cycles of 94°C for 30 s, 57°C for 30 s, and 72°C for 1.5 min, and a final cycle of 72°C for 10 min (for the nested reaction). All the primers and probes used in this study were ordered from Life Technologies (Grand Island, NY, USA).

### Amplification of positive-control Rickettsia.

We compared our nested 23S-5S IGS PCR assay with nested/seminested PCR assays employing three commonly used genes (*gltA*, *ompA*, and the 17-kDa protein gene) for detection of Rickettsia spp. in ticks. The PCR assays were carried out using genomic DNA for the above-mentioned 11 Rickettsia spp. A concentration of 2 ng gDNA from each positive-control sample was used as the template in primary PCR for all the PCR targets. The PCR mixture (20 μl) was comprised of 10 μl of reaction buffer and 1 μl each of forward and reverse primers (10 μM) with 1 μl of genomic DNA (2 ng) in the primary reactions, and 1 μl of the amplicon from the primary PCR was used as the template in subsequent nested PCR assays. Negative controls (reaction mixture with no DNA) were included in each set of PCRs. PCR primers used to amplify the 17-kDa protein gene (29), *ompA* (30, 31), and *gltA* (32, 33) and assay conditions used for the gene targets were described by Lee et al. (23). The amplicons, including 23S-5S IGS fragments, were subjected to electrophoresis on ethidium bromide-stained 1.2% agarose–Tris-acetate-EDTA (TAE) gels, and the banding patterns were visualized by UV transillumination. The amplicons were purified and subjected to DNA sequencing (Sanger) using the forward primer from the nested/seminested reactions. Two separate PCR assays were completed for each Rickettsia sp. for all four gene/IGS targets.

### DNA extraction from D. variabilis ticks.

Depleted adult ticks, collected from field sites in North Carolina by flagging vegetation (34), were preserved in 95% ethanol upon collection and stored at −20°C until they were processed. Genomic DNA was extracted individually from 40 D. variabilis adult ticks, using methods previously described (35). Crude DNA samples were purified with the Wizard DNA Clean-Up System (Promega, Madison, WI, USA), and the purified DNA was quantified with a NanoDrop (Thermo Scientific, Wilmington, DE, USA). All 40 tick genomic-DNA samples were normalized to a concentration of 75 ng/μl and stored at −20°C for later use.

### Comparison of the amplification efficiencies of PCR assays targeting Rickettsia in field-collected ticks.

Genomic-DNA samples from the 40 D. variabilis ticks that had been normalized to 75 ng/μl DNA were amplified for 4 Rickettsia genus-specific PCR targets (23S-5S IGS, *gltA*, *ompA*, and the 17-kDa protein gene). All the DNA samples were analyzed using the respective PCR thermocycling conditions outlined above, with two replicate (separate) assays completed for each tick/PCR target.

### Validation of 23S-5S IGS nested PCR assay. (i) Tick samples.

The new nested PCR assay was further validated using genomic DNA from 17 pools of D. variabilis ticks, which were tested previously for SFG rickettsiae by PCR targeting the *ompB* gene (10).

### (ii) Clinical samples.

In addition to ticks, three samples of gDNA from humans were tested using the nested 23S-5S IGS assay as described above. These samples were from a larger set of samples from clinical patients who were tested for R. rickettsii infection by Kato et al. to confirm RMSF (36). Similarly, blind tests were conducted using genomic-DNA samples from skin and organ tissues from laboratory animals (dogs, guinea pigs, goats, and rabbits) that had been challenged with R. rickettsii (37) or *R*. slovaca (G. Zemtsova, unpublished studies). Also, genomic DNA from skin, blood and other organ tissue samples (lung, liver, spleen, kidney, and lymph node) taken from some of the laboratory animals were extracted in our laboratory (35) and analyzed for Rickettsia using the new PCR assay. All the clinical samples were tested twice in separate PCR assays, each using 2.5 μl of template DNA in the primary cycle and 1 μl of the primary amplicon in the subsequent nested cycle. Zemtsova et al. originally tested the samples from laboratory animals that were experimentally infected with Rickettsia using a SYBR green-based real-time PCR with 5 μl of template DNA (37). 23S-5S IGS fragments amplified from clinical samples were sequenced as described below to confirm the identity of the Rickettsia spp.

### Quality control of PCR assays.

All tick and clinical tissue samples were processed in a nonventilated PCR enclosure. PCRs were prepared in the nonventilated PCR enclosure or a laminar flow hood, which were located in separate rooms. On each occasion, prior to being used, the work areas were thoroughly cleaned with ethanol and exposed to UV light. Every PCR run included positive-control (DNA from R. parkeri) and negative-control (PCR mixture but no template DNA) samples.

### Establishing the limits of detection.

Plasmid DNA ligated with targeted PCR fragments of “Candidatus Rickettsia amblyommii” was used to establish the limits of detection (38). Briefly, 23S-5S IGS, *ompA*, and *gltA* PCR fragments of “Candidatus Rickettsia amblyommii” were cloned separately using the pGEM-T Cloning kit (catalog no. A1360; Promega, Madison, WI, USA). Individual white colonies from each plate were picked and grown overnight at 37°C in LB broth supplemented with ampicillin (100 μg ml^−1^). Plasmid DNA from each overnight culture was extracted using an UltraClean Standard Mini Plasmid Prep kit (catalog no.12301; Mo Bio Laboratories Inc., CA, USA). The identities of inserts from the plasmid DNA were confirmed by amplifying them with vector primers M13F (5′ CCCAGTCACGACGTTGTAAAACG 3′) and M13R (5′ AGCGGATAACAATTTCACACAGG 3′) and sequencing and analyzing the DNA fragments using BLASTN (39). Subsequently, primary and nested PCR assays of the 3 targets were conducted, using their respective protocols (as described above), on 10-fold serial dilutions of plasmid DNA ranging from 10^6^ to 1 copy per μl. The copy number in the dilutions was calculated using the DNA copy number calculator on the Life Technologies website. To determine if tick genomic DNA interfered with the amplification of Rickettsia DNA, a series of plasmid DNA dilutions, each spiked with 75 ng of D. variabilis DNA that was negative for Rickettsia DNA, were also tested concurrently.

### RLB hybridization.

RLB hybridization was conducted using ∼350-bp 23S-5S IGS nested amplicons of Rickettsia DNA from positive controls and D. variabilis ticks as described by Lee et al. (23). Briefly, amplicons from 23S-5S IGS nested PCR assays displaying a band of the expected size on agarose electrophoresis gels were diluted by mixing 10 μl of the PCR product and 180 μl of 2× SSPE (1× SSPE is 0.18 M NaCl, 10 mM NaH_2_PO_4_, and 1 mM EDTA [pH 7.7])-0.1% SDS solution and used in RLB hybridization assays. The hybridization temperature was 52°C for 1 h, and the stripping solution concentration and temperature were 0.5% SDS and 60°C, respectively. Hybridized PCR products were detected by chemiluminescence with a ChemiDoc-ItTS2 imaging system (UVP, Upland, CA, USA) following incubation of the membrane in ECL detection liquid (Amersham, Little Chalfont, Buckinghamshire, United Kingdom).

### Sequencing and phylogenetic analyses.

Nucleotide sequence analysis of the 23S-5S IGS fragments was used to confirm the identity of the Rickettsia spp. used as positive controls and to identify the Rickettsia spp. in the D. variabilis DNA samples that were analyzed by RLB hybridization. Attempts were also made to sequence amplified DNA from the other three PCR targets. The forward primers used in the nested PCR were employed for sequencing Rickettsia spp. (23-5SN1F [23S-5S IGS], 190.70p [*ompA*, 5′ ATGGCGAATATTTCTCCAAAA 3′] RpCS896p [*gltA*, 5′ GGCTAATGAAGCAGTGATAA 3′], and 17kN1F [the 17-kDa protein gene, 5′ CATTACTTGGTTCTCAATTCGGT 3′]), which was performed by Eton BioScience, Inc. (Research Triangle Park, NC, USA). DNA sequences were identified by searching (BLASTN) on the partial Rickettsia sequences in the NCBI database.

The 23S-5S IGS sequences from the positive-control Rickettsia spp. and ticks were aligned with sequences deposited in GenBank using Clustal X version 2.0 (27), and the sequences were trimmed at both ends to an average length of 330 bp. Phylogenetic analysis was conducted by the neighbor-joining method (40), using the Kimura two-parameter model with partial gap deletion and a cutoff of 95% site coverage. The evolutionary distance was calculated, and bootstrap analysis with 1,000 iterations was carried out with the MEGA6 software package (28).

### Nucleotide sequence accession numbers.

The 23S-5S IGS sequences of the known Rickettsia spp. generated in this study were submitted to GenBank (accession no. KT340601 to KT340611). The 23S-5S IGS, *gltA*, and *ompA* sequences from D. variabilis ticks were also deposited in GenBank (accession no.: 23S-5S IGS, KT374186 to KT374204; *gltA*, KT374205 to KT374212; *ompA*, KT374213 to KT374216).

## RESULTS

### Amplification of positive-control Rickettsia spp. and limits of detection.

Attempts were made to amplify four PCR targets (the 17-kDa protein gene, *ompA*, *gltA*, and 23S-5S IGS) from 11 Rickettsia species, including members of the SFG and TG, R. bellii, and R. canadensis (Table 1). The 23S-5S IGS nested method amplified all Rickettsia spp., producing a band of the expected size, ∼350 bp. The identities of 23S-5S IGS nested amplicons of 11 positive controls were confirmed by sequencing and by aligning them to known Rickettsia sp. sequences from GenBank. At the same DNA concentration, the PCR targeting *ompA* amplified all the SFG rickettsiae, but not *R*. bellii, R. canadensis, or R. typhi. The *gltA* assay amplified all but R. bellii, and the 17-kDa protein gene assay did not produce visible bands for R. bellii and R. canadensis. Failure to amplify *gltA* of *R*. bellii appeared to be due to mismatches of reverse primers for the primary (4 of 22 nucleotides) and nested (3 of 19 nucleotides) amplifications with the target gene sequence (GenBank accession no. DQ146481.1).

Amplifications of serially diluted plasmid DNA ligated with 23S-5S IGS, *ompA*, and *gltA* amplicons of “Candidatus Rickettsia amblyommii” were tested separately to compare their levels of detection. As little as 1 copy of the 23S-5S IGS fragment in the primary PCR was enough to produce a highly visible gel band in the nested PCR. The sensitivity of the *ompA* gene was on par with that of the 23S-5S IGS; however, the sensitivity of *gltA* gene amplification was 10-fold lower than that of the other two PCR targets. The amplification sensitivity decreased by one 10-fold dilution in the presence of tick DNA for all three PCR targets. Serial-dilution assays for the three targets were repeated twice in separate PCR amplifications.

### Validation of the 23S-5S IGS PCR assay. (i) Tick samples.

Seventeen D. variabilis DNA samples that were previously analyzed for the Rickettsia *ompB* gene in another laboratory were used to validate our 23S-5S IGS nested assay. Seven out of 17 samples were reported to be positive for R. montanensis, and 10 samples were PCR negative. With the 23S-5S IGS nested assay, all 7 positive samples and 2 of the 10 *ompB* gene-negative samples produced bands of the expected size (∼350 bp) on agarose gels (data not shown). The two samples that failed to amplify in PCR assays targeting the *ompB* gene but that tested positive in our 23S-5S IGS nested assay were subsequently sequenced and identified as R. montanensis.

### (ii) Clinical samples.

Clinical samples from humans and laboratory animals were used to further validate the nested 23S-5S IGS assay. All three DNA samples from humans known to be infected with R. rickettsii tested positive using the nested assay based on visual detection of bands of the expected size on agarose electrophoresis gels. These amplicons were sequenced and found to be 99 to 100% homologous to R. rickettsii (GenBank accession no. CP006010.1). The results of the blind tests of samples from laboratory animals carried out with the new nested PCR assay were highly concordant with those of the real-time PCR tests. The 23S-5S IGS nested assay detected Rickettsia DNA in 88% (15 of 17) of the gDNA samples that were reported positive when tested by real-time PCR. For the tissue samples extracted in our laboratory, the 23S-5S IGS nested assay detected DNA of Rickettsia in 4 of 5 samples that were positive by real time-PCR. Notably, the nested 23S-5S IGS assay detected Rickettsia DNA in one sample that was reported to be negative by real-time PCR. The majority (12 of 17) of Rickettsia-positive tissue samples were from skin, but skin comprised only 65% (13 of 20) of the total number of tissue samples tested. Rickettsia DNA was detected in skin samples resected at the site of the tick bite, as well as from distally located sites. Generally, rickettsiae were not detected in organ tissue samples from the spleen, heart, kidney, or lung. The Rickettsia spp. in laboratory animal clinical samples were 99 to 100% homologous to partial 23S-5S IGS sequences of R. rickettsii (accession no. CP006010.1) and *R*. slovaca (accession no. CP003375.1) deposited in GenBank.

### Comparison of the amplification efficiencies of PCR assays targeting Rickettsia in field-collected ticks.

**FIG 1:**
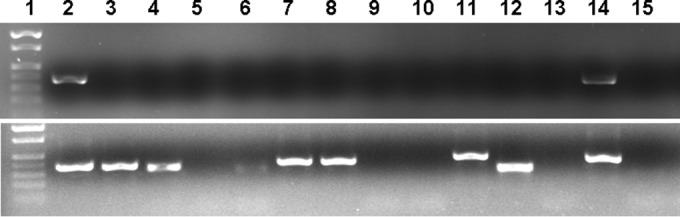
Representative PCR amplification of Rickettsia 23S-5S IGS from genomic-DNA samples of D. variabilis ticks. Primary (top) and nested (bottom) amplifications are shown. Lanes 1, marker; lanes 2, R. conorii positive control; lanes 3 to 15, representative tick samples. Rickettsia-positive samples are shown in lanes 3, 4, 7, 8, 11, and 14. The amplicon in lane 12 is not of Rickettsia origin.

**FIG 2:**
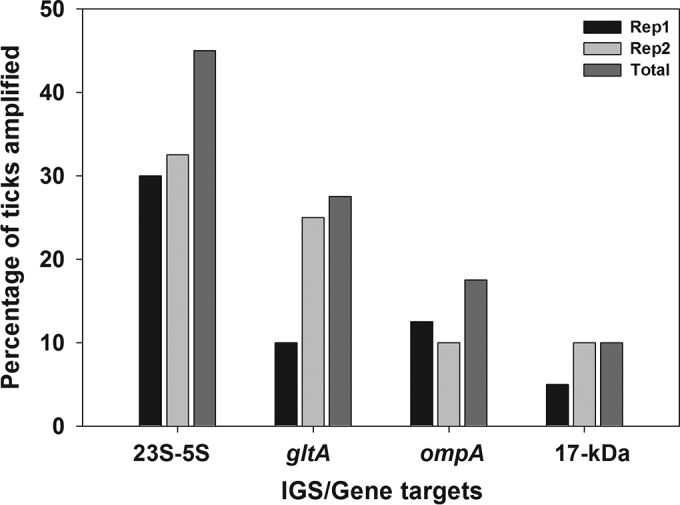
Comparative detection rates of Rickettsia spp. for nested/seminested PCR assays for four gene/IGS targets. Amplification success is expressed as a percentage of 40 D. variabilis ticks tested. The total percentage was calculated using the sum of the number of ticks that were PCR positive in either or both replicate assays (Rep1 and Rep2) as the numerator.

Attempts were made to amplify Rickettsia spp. from 40 D. variabilis DNA samples with the 4 PCR targets. After the primary 23S-5S IGS amplification, only 2 out of the 40 samples (5%) showed bands of the expected size in agarose electrophoresis gels in both replications (Fig. 1), but the detection rate increased to 45% (18/40) after the nested step in the assay. The other PCR assays amplified only between 5 and 20% of the samples after the secondary PCR amplification (Fig. 2).

### Reverse line blot hybridization and nucleotide sequence analysis.

A reverse line blot hybridization assay was used for high-throughput identification of 23S-5S IGS nested amplicons from D. variabilis ticks. All control DNAs hybridized with the Rickettsia genus probe (GP-RICK) and also hybridized with the intended DNA probes specific for SFG and TG species of Rickettsia. All 18 of the 23S-5S IGS-positive samples from 40 D. variabilis samples hybridized to the Rickettsia genus-specific probe. The samples predominantly hybridized to the “Candidatus Rickettsia amblyommii”-specific probe but also to probes for R. conorii, *R*. montanensis, *R*. bellii, and *R*. massiliae. Some unknown species of Rickettsia and SFG species were also detected (Table 2). The RLB results were further evaluated by nucleotide sequencing of 23S-5S IGS amplicons (Table 2). BLAST results for the 23S-5S IGS sequences showed high (99 to 100%) similarity to “Candidatus Rickettsia amblyommii,” R. montanensis, R. bellii, and R. felis and the presence of some potentially novel Rickettsia species. Nucleotide sequence analysis was generally congruent with the RLB hybridization results (Table 2). Concurrently, amplicons from the other PCR targets (*gltA*, *ompA*, and the 17-kDa protein gene) that produced visible bands on agarose gels were also sequenced, and the sequences showed the highest similarity to R. montanensis and “Candidatus Rickettsia amblyommii”.

**TABLE 2:** PCR amplification success of Rickettsia 23S-5S IGS fragments from 18 D. variabilis adult ticks and comparison of the identification of Rickettsia spp. through Sanger sequencing versus RLB hybridization of the same 23S amplicons

Sample identifier code	Amplification success for replicate^*a*^		Nucleotide sequence identification for replicate^*b*^		RLB hybridization identification
	1	2	1	2	
29-4	+	−	“Candidatus Rickettsia amblyommii”	−	“Candidatus Rickettsia amblyommii”
32-1	+	+	“Candidatus Rickettsia amblyommii”	R. bellii	“Candidatus Rickettsia amblyommii,” R. massiliae
33-3	+	+	“Candidatus Rickettsia amblyommii”	“Candidatus Rickettsia amblyommii”	“Candidatus Rickettsia amblyommii”
38-2	+	−	R. conorii	−	R. conorii
39-10	+	+	R. bellii	R. rhipicephali	R. bellii
44-10	+	+	−	R. felis	Unknown SFG
44-30	−	+	−	R. conorii	R. conorii
44-35	−	+	−	“Candidatus Rickettsia amblyommii”	“Candidatus Rickettsia amblyommii”
44-45	+	−	−	−	R. conorii
44-50	+	+	R. felis	R. bellii	Unknown SFG
44-55	+	+	“Candidatus Rickettsia amblyommii”	“Candidatus Rickettsia amblyommii”	“Candidatus Rickettsia amblyommii”
44-65	+	+	R. montanensis	R. montanensis	R. montanensis
44-85	−	+	−	“Candidatus Rickettsia amblyommii”	“Candidatus Rickettsia amblyommii”
44-90	−	+	−	R. montanensis	Unknown SFG
44-95	+	−	“Candidatus Rickettsia amblyommii”	−	“Candidatus Rickettsia amblyommii”
45-4	−	+	−	“Candidatus Rickettsia amblyommii”	“Candidatus Rickettsia amblyommii”
48-1	+	+	R. montanensis	R. montanensis	R. montanensis
52-2	+	−	R. conorii	−	R. conorii

a +, PCR amplicon was detected on the agarose gel; −, PCR amplicon was not detected on the agarose gel.

b −, multiple sequences; coinfection with >1 Rickettsia sp.

### Phylogenetic analysis.

**FIG 3:**
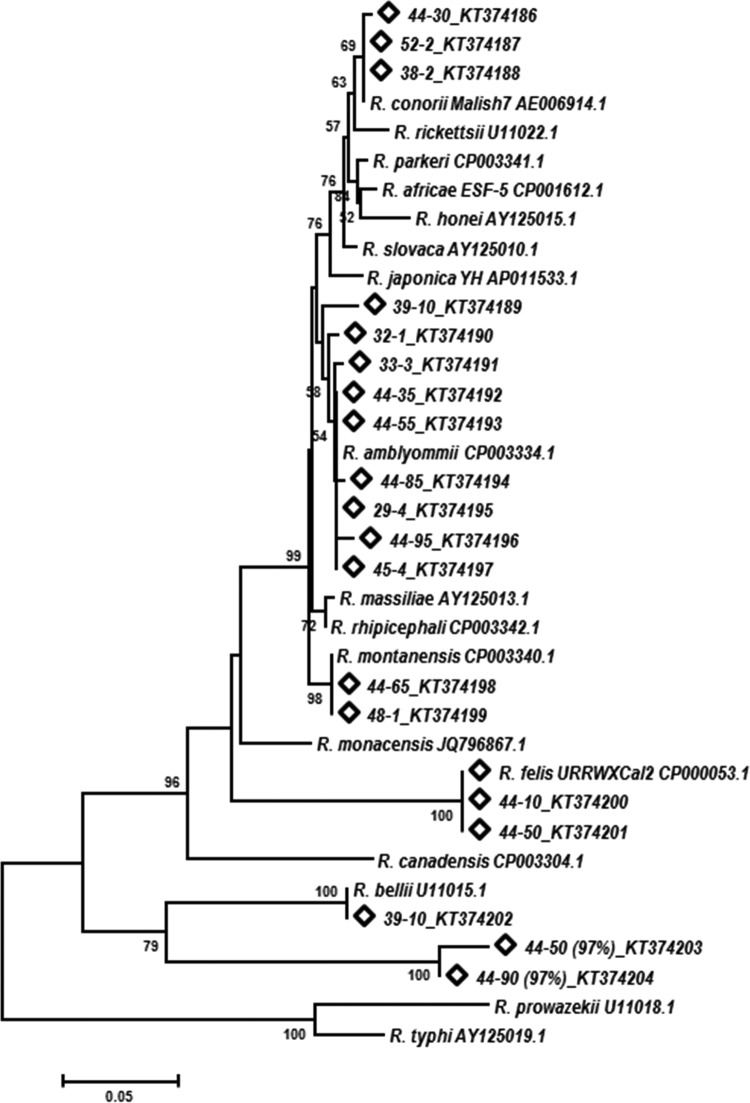
Neighbor-joining tree showing phylogenetic relationships of partial 23S-5S IGS sequences of known Rickettsia species taken from the NCBI database and sequences amplified from D. variabilis ticks. The scale bar indicates an estimated change of 5% 23S-5S IGS. The sequences marked with diamonds were generated in this study. Bootstrap values below 50% are not shown in the tree branches.

Phylogenetic analysis of Rickettsia spp. using the neighbor-joining method showed that D. variabilis ticks contained a diverse group of rickettsiae. Potentially novel Rickettsia spp. with 97% similarity to R. bellii were placed on a separate branch. Multiple ticks contained a Rickettsia species that was placed on the same branch as R. conorii. Rickettsiae identified through sequence match analysis as “Candidatus Rickettsia amblyommii,” *R*. montanensis, and R. felis in this study were aligned with these species in the phylogenetic tree (Fig. 3).

## DISCUSSION

Our novel nested 23S-5S IGS PCR assay was shown to be a robust molecular tool for detecting Rickettsia species. Developing new diagnostic tools and improving the existing ones facilitates molecular epidemiological investigations of tick-borne diseases (17). In this regard, the new nested assay should find utility in detecting Rickettsia spp. in ticks and clinical samples. Pathogen surveillance in field-collected ticks by PCR amplifying specific gene targets is a widely used practice for determining the prevalence and diversity of the genus Rickettsia in a given environment (8, 19–21, 24, 41). We found that our nested 23S-5S IGS PCR assay outperformed the 17-kDa protein gene, *ompA*, and *gltA*, which are commonly used gene targets in molecular surveys of rickettsiae in ticks and other environmental samples. In light of the increasing number of cases of SFG rickettsiosis (4), which could potentially be caused by new Rickettsia spp. (4, 6), it is essential to develop new and/or to improve existing molecular assays to increase the detection rate of SFG and other rickettsiae vectored by ticks and other blood-feeding arthropods (17).

The new nested 23S-5S IGS assay amplified all 11 positive-control Rickettsia spp. representing the SFG, TG, and ancestral groups. In contrast, at the same template gDNA concentration, other gene targets failed to amplify all the phylogenetic groups of Rickettsia, making 23S-5S IGS a robust target for pan-Rickettsia detection. The utility of a conventional PCR assay targeting the Rickettsia 23S-5S IGS was reported previously (22); however, adding a nested step substantially improved the sensitivity of the assay. The highly conserved ends and the hypervariable central regions make it an ideal target for understanding the phylogenetic relationships of SFG and other species of Rickettsia (2, 25). The greater efficiency of 23S-5S IGS for Rickettsia detection compared to other PCR targets is likely due to greater overall coverage and primer specificity across the genus. The occurrence of the 23S-5S IGS in the SFG and the typhus and ancestral rickettsial groups is a major strength of our newly developed assay.

The American dog tick (D. variabilis) is an established vector of RMSF. However, recent studies have generally reported that ≤10% of ticks were infected with SFG rickettsiae, and R. rickettsii, the causal organism of RMSF, was rarely detected (13, 42–45). The newly developed 23S-5S IGS PCR assay used in this study detected DNA of Rickettsia spp. in 18/40 (45%) D. variabilis ticks tested, while other PCR assays amplified Rickettsia spp. from 5 to 20% of the same samples of tick gDNA. In our nested PCR assays for the Rickettsia 23S-5S IGS, 7 of 18 tick samples were positive in both replicates. Similarly, we observed differences in the patterns of sample positivity for the two replications of the 17-kDa protein gene, *ompA*, and *gltA* gene targets. Generally, for all PCR targets, samples that produced visible bands in the primary PCR were positive in both replications. These findings suggest that the low abundance of Rickettsia DNA in some tick genomic-DNA samples may have affected amplification regardless of the PCR target.

While we did not detect R. rickettsii in any of the D. variabilis ticks that were screened, we detected DNA of an R. conorii-like species in some ticks. R. conorii is the pathogen causing Mediterranean spotted fever (3) and is closely related to R. rickettsii. To our knowledge, this Rickettsia species has not been reported to occur in North America, and additional studies will be required to further characterize the organism. Interestingly, the R. conorii-like 23S-5S IGS fragment amplified in the present study from D. variabilis is 99 to 100% homologous in nucleotide sequence to a 23S-5S IGS fragment amplified from Amblyomma americanum Linn. in our previous study (23). This finding suggests that these ticks might have become infected with the rickettsia by feeding on the same vertebrate host species.

RLB hybridization assay of the 23S-5S IGS nested amplicons identified diverse Rickettsia spp. in D. variabilis ticks. The PCR-RLB assay has been shown to be a sensitive and specific assay for detection and identification of pathogenic and nonpathogenic Rickettsia spp. (22, 23, 46, 47). Notably, adding a nested step in the PCR amplification of the 23S-5S IGS fragments substantially improved the sensitivity of the assay for detection of rickettsiae at low abundance. The phylogenetic analysis of the 23S-5S IGS sequence data confirmed the presence of potentially novel Rickettsia species in D. variabilis ticks. Use of the nested 23S-5S IGS PCR-RLB hybridization assay for high-throughput screening of additional field-collected D. variabilis adults will be reported in a subsequent publication.
